# Interpretation Biases in Pain: Validation of Two New Stimulus Sets

**DOI:** 10.3389/fpsyg.2021.784887

**Published:** 2022-01-06

**Authors:** Daniel Gaffiero, Paul Staples, Vicki Staples, Frances A. Maratos

**Affiliations:** Department of Health, Psychology and Social Care, University of Derby, Derby, United Kingdom

**Keywords:** adults, cognitive biases, ambiguous scenarios, pain, interpretation bias

## Abstract

Adults with chronic pain interpret ambiguous information in a pain and illness related fashion. However, limitations have been highlighted with traditional experimental paradigms used to measure interpretation biases. Whilst ambiguous scenarios have been developed to measure interpretation biases in adolescents with pain, no scenario sets exist for use with adults. Therefore, the present study: (i) sought to validate a range of ambiguous scenarios suitable for measuring interpretation biases in adults, whilst also allowing for two response formats (forced-choice and free response); and (ii) investigate paradigm efficacy, by assessing the effects of recent pain experiences on task responding. A novel ambiguous scenarios task was administered to adults (*N* = 241). Participants were presented with 62 ambiguous scenarios comprising 42 that could be interpreted in a pain/pain-illness or non-pain/non-pain illness manner: and 20 control scenarios. Participants generated their own solutions to each scenario (Word Generation Task), then rated how likely they would be to use two researcher-generated solutions to complete each scenario (Likelihood Ratings Task). Participants also rated their subjective experiences of pain in the last 3 months. Tests of reliability, including inter-rater agreement and internal consistency, produced two ambiguous scenario stimulus sets containing 18 and 20 scenarios, respectively. Further analyses revealed adults who reported more recent pain experiences were more likely to endorse the pain/pain-illness solutions in the Likelihood Ratings Task. This study provides two new stimulus sets for use with adults (including control items) in pain research and/or interventions. Results also provide evidence for a negative endorsement bias in adults.

## Introduction

19% of Adult Europeans experience moderate to severe Chronic Pain (Breivik et al., 2006). Theoretical models of pain assert that cognitive biases play an important role in the etiology and maintenance of chronic pain (Van Ryckeghem et al., 2019). For example, much research indicates adults with chronic pain display negative interpretation biases (IB) for pain/illness related information (e.g., Schoth and Liossi, 2016; Schoth et al., 2019; Chan et al., 2020). Interpreting ambiguous information in a pain-related manner is thought to contribute to the development and maintenance of chronic pain via increased pain catastrophizing and fear of pain (Khatibi et al., 2014, 2015), both of which promote fear-avoidance behaviors (Buer and Linton, 2002; Andersen et al., 2016) that actively discourage individuals from undertaking everyday activities that promote recovery (e.g., exercise), contributing to increased disability (Elfving et al., 2007; Gheldof et al., 2010). Indeed, theoretical models of pain, including the Threat Interpretation Model (TIM, Todd et al., 2015) posit that the interpretation of a stimulus as pain-relevant and threatening are pre-requisites for attentional biases (AB, i.e., the allocation of attentional resources toward pain-related over neutral stimuli in one’s environment) to be observed. Hence, the investigation of IB in pain-related research is of critical importance.

Interpretation biases s have typically been investigated using a variety of experimental paradigms, which has raised concerns surrounding the methodological heterogeneity of IB research given the variance in the use of experimental stimuli and assessment methods including direct (e.g., written response) and indirect (response time) measures (for full review see Schoth and Liossi, 2017). Generally speaking, IB paradigms can be organized into three main categories; ***single ambiguous words***; including the Homographic Response Task (McKellar et al., 2003), Homophone Task (e.g., Pincus et al., 1996), Sentence Generation Task (Taghavi et al., 2000; e.g., Schoth et al., 2018, 2019) and Word-stem Completion Task (e.g., Edwards and Pearce, 1994; Griffith et al., 1996); ***ambiguous images***; including the Incidental Learning Task (Khatibi et al., 2014, 2015); and ***ambiguous scenarios***; including the Ambiguous Scenarios Test (e.g., Heathcote et al., 2016, 2017; Lau et al., 2019). These paradigms will be briefly described and evaluated below.

With respect to single ambiguous words, Homographs, Homophones, Word-Stem Completion and Sentence Generation Tasks have all been used in pain-related IB research. In the Homographic Response Task written homographs (e.g., Sharp – ***clever***, Sharp – ***pain***) are presented individually. Participants are then instructed to note the first word that enters their mind relating to each homograph. Independent judges categorize these responses as pain-related, disability-related or neutral. In the Homophonic Response Task, spoken homophones with pain-related (e.g., Pain) and neutral associations (e.g., Pane) are presented. Participants are instructed to note down their interpretation of that word. Here, IB is quantified by the number of homophones interpreted in a pain-related versus neutral manner. In the Sentence Generation Task homographs are presented individually (e.g., Sharp), participants are then asked to integrate the homophone into a sentence. Independent judges categorize each sentence as pain-related (i.e., using the homograph in a pain/pain-illness related manner, e.g., He felt a ***sharp*** pain in his leg) or benign (using the homograph in a non-pain/non-pain illness related manner, e.g., He has a very ***sharp*** mind). Finally, the word-stem completion task involves the presentation of a word stem (e.g., Ten_ _ _) that can be interpreted in a pain/illness-related (Ten***der***) or non-pain/non-pain illness related manner (Ten***nis***). Participants complete the word stem using the first word that enters their mind. A higher number of pain/illness-related word completions is indicative of a negative pain-related IB.

While these paradigms are all easy to administer and offer relatively straightforward response classifications, in many instances the stimuli used restrict response selection choice. For example, homophones have differences in verbal and written frequencies of use (e.g., “Pain” has a higher written and verbal frequency than “Pane”). Thus, the likelihood of observing between-groups differences is reduced, irrespective of pain suffering, as most would interpret this homophone as “pain” due to its higher daily life frequency. Equally, the extent to which these paradigms suffer from demand characteristics and are influenced by other stimulus-related factors (e.g., word length) has also come under scrutiny (Schoth and Liossi, 2017). Hence, the utility of these paradigms in appropriately measuring IBs has been questioned.

The tasks discussed above are all consistent in that they measure IB directly via written responses. That said, indirect measures of IB (using response time) have also been developed including the Incidental Learning Task (see Khatibi et al., 2014, 2015) which makes use of morphed facial expressions. This paradigm involves two distinct phases, a learning phase and a test phase. In the learning phase a facial expression (e.g., Pain, Happy) is presented on a computer screen centrally. The type of facial expression displayed is predictive of the location of a subsequent target cue (e.g., Pain = upper target, Happy = lower target). During the test phase, a neutral facial expression is presented with targets appearing randomly (but with equal frequency) at upper/lower locations. Here, IB is quantified by measuring the amount of time taken for participants to respond to target cues predictive of specific facial expressions (e.g., Pain or Happiness). A key strength of this paradigm is that it avoids the demand characteristics associated with single ambiguous words, and offers an indirect means of measuring IB. However, the use of morphed facial expressions has been criticized for possessing lower ecological validity (than standard facial expressions) due to appearing unnatural and unlike facial expressions viewed in normal life (Schoth and Liossi, 2017).

Most recently, paradigms using Ambiguous Scenarios have been developed to measure pain related IBs. Whilst multiple versions exist within the broader IB literature (e.g., Ambiguous Scenarios Test), Heathcote et al. (2016) developed the Adolescent Interpretation of Bodily Threat Task (AIBT) to more rigorously explore pain related IB in youth. The AIBT contains eight scenarios describing ambiguous situations interpreted as relating to bodily threat or pain. Participants imagine themselves in the scenarios and are then offered two solutions that resolve each in a negative or benign manner (e.g., “You see a boy breathing heavily. His chest is quickly going up and down. He is…” Asthmatic/Exercising). Also presented are eight ambiguous social situation scenarios (e.g., “Your school is looking for a new person to join their debating team. You ask for more details. After hearing these, you decide you would be…” Rejected/Welcomed). They then rate whether each interpretation was “likely to enter their mind,” and which solution “most likely came to mind,” as well as their belief that each “interpretation is a true reflection of reality.” Developing this research, Lau et al. (2019) doubled the AIBT stimulus set to 32 items (16 - bodily harm/16 - social situations). They further adjusted the response format, with participants reporting the degree to which each negative/benign interpretation is likely to explain a situation. Both studies found evidence that adolescents experiencing pain were more likely to display a negative IB, highlighting the utility of ambiguous scenarios in measuring IBs.

However, one limitation of the AIBT task is that it constrains participants to a set of pre-determined interpretations (i.e., forced-choice responses), thus, the solutions offered to participants may not reflect their own personal interpretation of each scenario. Further, the scenarios currently in use are only validated with adolescent populations, with many items not appropriate for investigating IB in adults. Developing age-appropriate stimuli is therefore of critical importance. Consequently, we sought to validate a range of ambiguous scenarios suitable for use with adult populations in pain research/treatment interventions; and allowing for two response formats (forced-choice and free response). Participants were presented with a Word Generation (free response) and Likelihood Ratings (forced choice) task. Pain experiences in the preceding 3 months were measured to assess effects of pain experiences on task responding.

## Materials and Methods

### Participants

Participants were recruited via the distribution of a study advertisement. This stated inclusion criteria of fluency in English, normal or corrected-to-normal vision and age (18 or over); and resulted in recruitment of an opportunity sample of 521 participants from the local United Kingdom University and wider United Kingdom (general) population. However, 278 participants were excluded from analysis due to providing incomplete responses. A further two participants were excluded as a result of violating the age-related inclusion criteria. Thus, the final sample compromised 241 participants, including 55 males (23.23%), 181 females (74.68%) and 5 who preferred not to declare their gender (2.07%). The age of participants ranged from 18 to 79 years (*M* = 28.88, *SD* = 10.83). For compensation of their time and commitment to the study, students (24.06%) received course credit. Participants from the wider population were entered into a prize draw to win a £20 Amazon Voucher. The study was approved by the local Human Sciences Research Ethics Committee and informed consent gained from each participant prior to participation.

### Design

The study (conducted online, to encourage a wide variety of demographics) employed a repeated measures design. The Independent Variable (Scenario Type) had two levels; Ambiguous and Control. Participants completed two tasks; a Word Generation Task and Likelihood Ratings Task for both ambiguous and control scenarios. These tasks were not counterbalanced to avoid priming participants. To expand, solutions provided in the Likelihood Ratings Task (Pain/Pain-Illness and/or Non-Pain/Non-Pain Illness) could have inadvertently influenced responding to the Word Generation Task. Hence, the Word Generation Task was completed first by all participants.

### Word Generation Task

In the word generation task, participants were presented with one of the ambiguous or control scenarios in the center of the screen in a randomized order. For example:

‘Your Dad leaps up from his chair making a loud noise. He is…’

    
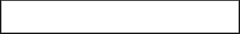


Participants were instructed to type a response in the box using the first word (or words) that came to mind. Each scenario was presented in the center of the screen in 11.5 sized “Helvetica” font. Once participants had provided responses to all 62 scenarios the task was complete.

### Likelihood Rating Task

In the likelihood rating task, participants were presented with one of the ambiguous or control scenarios in the center of the screen in a randomized order. This time, however, two-word solutions appeared simultaneously. For pain/pain-illness scenarios, one pain or illness solutions and one non-pain/non-pain illness related solution appeared with the ambiguous scenario. For example:

“You drop a kitchen knife on the floor. It … your foot.”

              Cuts

            Misses

For control scenarios, two non-pain/non-pain illness related solutions appeared with the ambiguous scenario. For example:

“You arrive at the office to start the working day. You turn on the…”

            Computer

               Lights

Next, similar to the methodology of Heathcote et al. (2016) participants were required to indicate how likely they would be to use each solution to complete the scenario by assigning a likelihood percentage using a sliding scale ranging from 0 to 100%. As participants were asked to rate likeliness for each solution on a 0-100% scale, ratings were not mutually exclusive (i.e., if participants rated their likelihood of using the first solution to complete the scenario as 70%, they were not restricted to rating the second solution as 30% likely to complete the scenario). Each scenario was presented in the center of a new screen in 11.5 sized ‘Helvetica’ font. Once likelihood ratings had been provided for the two solutions for each of the 62 scenarios, the task was complete.

### Materials

#### Stimulus Set Creation

A stimulus set comprising 62 ambiguous scenarios was collated. Of these 42 were stimuli that would elicit variability among participants in terms of pain/pain-illness vs. non-pain/non-pain illness interpretations and 20 were designed to elicit variability in terms of only non-pain/non-pain illness interpretations.

##### Pain/Pain-Illness vs. Non-pain/Non-pain Illness Scenarios

Of the **42** scenarios produced, **12** were sourced from previous research (Heathcote et al., 2016, 2017; Lau et al., 2019). The remaining **30** scenarios were generated by authors DG, FM, and PS. This involved an iterative process of each author generating scenarios and those judged by all three as ambiguous (in that they could be interpreted in a pain/pain-illness ***or*** non-pain/non-pain illness manner), added to the 12 scenarios sourced from previous research. In example:

“You drop the kitchen knife onto the floor, it … your foot.”

This scenario is ambiguous because there are at least two potential responses that reflect different interpretations. For instance, the word “**hits”** would reflect a pain-related interpretation and “**misses”** would indicate a non-pain related interpretation.

##### Control Scenarios

The above process was repeated to further generate a set of entirely novel control scenarios to act as “filler” stimuli to avoid demand characteristics or priming participants with the ambiguous scenarios. Although, as for the ambiguous scenarios, the same three authors first generated many control scenarios and then selected scenarios on the basis that all agreed they appeared ambiguous but, importantly, non-pain/non-pain illness related. This resulted in 20 such scenarios. For example:

“Your partner was late to an important meeting. This is because they forgot their…”

This scenario is ambiguous as there are at least two potential responses, such as the words “**phone”** and “**keys**.**”** However, this scenario is also non-pain/non-pain illness related in that potential responses are very unlikely to reflect a pain/pain illness interpretation.

Therefore, in total, the study comprised of **62** scenarios. Of which, **42** were “**ambiguous”** but potentially pain/illness related and **20** were “**control”**; that is, not pain nor pain-illness related. The mean number of words of each scenario in the ambiguous (*Md* = 15, *n* = 42) and control (*Md* = 14, *n* = 20) categories was matched/controlled for (*p* = *0.431*).

#### Questionnaires

##### Recent Pain Experiences Questionnaire (RPEQ)

To assess participant’s subjective experiences of pain in the last 3 months, four items were derived from the Brief Pain Inventory Short-Form (Cleeland and Ryan, 1994). Consistent with previous research, using an 11-point Likert scale (0–10), participants were required to rate their: (i) average pain intensity; (ii) worst pain intensity; (iii) the amount that pain had interfered with daily activities; and (iv) the frequency of their pain (Heathcote et al., 2016; Said et al., 2019). For each item, scores can range from 0-10, with higher scores indicating a higher average pain intensity, worst pain intensity, pain interference with daily activities and frequency of pain, respectively. The Brief Pain Inventory has been shown to be both reliable and valid across many cultures and languages (Cleeland and Ryan, 1994), and in the measurement of pain in numerous conditions including chronic non-malignant pain (Antony et al., 1998), osteoarthritis (Kapstad et al., 2010) and cancer pain (Kumar, 2011).

##### Depression, Anxiety and Stress Scale (DASS-21)

To ascertain the endorsement of solutions was linked to pain as opposed to generalized anxiety/depression symptomology, the DASS-21 (Henry and Crawford, 2005) was used. This is important as it enables us to assess whether one’s experience of pain, as opposed to any anxiety/depression symptomology, influences the biased interpretation of ambiguous information. The DASS-21 is a 21-item questionnaire, comprised of 3 sub-scales of 7 items each: depression, anxiety and stress. Participants are required to rate each item on a 4-point Likert-type scale ranging from 0 (does not apply to me at all) to 3 (applied to me very much, or most of the time). Total sub-scale scores can range from 0 - 21, with higher scores indicating increased severity of depression, anxiety and/or stress, respectively. To enable comparison with the original DASS-42 scale, total sub-scale scores are doubled and thus can range from 0 – 42. Research has tested the psychometric properties of the DASS-21 and found each sub-scale possesses adequate internal consistency, concurrent validity and very good Cronbach’s alpha; values of.84, 0.74, and 0.79 for depression, anxiety and stress, respectively (Antony et al., 1998; Musa et al., 2007; Asghari et al., 2008; Wood et al., 2010).

#### Procedure

The study was designed and completed using Qualtrics (Provo, UT). Participants were instructed that in order to participate they would need to complete the study individually in a quiet location and were required to confirm such conditions. Once confirmed and informed consent gained, participants provided demographic information then completed the Word Generation Task followed by the Likelihood Ratings Tasks. Once participants had completed both scenario tasks, they then completed the *RPEQ* and the *DASS-21* questionnaires prior to being presented with a debrief. This included signposting to relevant support organizations in case of concerns (i.e., counseling helplines, pain concern). On average, the online study took participants 45 min to complete.

## Results

### Participant Characteristics

Descriptive data is presented in Table 1. A Mann-Whitney U test indicated no significant sex differences in depression (*p* = 0.08), anxiety (*p* = 0.10), stress (*p* = 0.93) or frequency of pain (*p* = 0.77). However, significant sex differences were observed for ratings of average pain (*U* (N_*Males*_ = 55, N_*Females*_ = 181) = 3671.5, *z* = −2.99, *p* < 0.01), with females reporting more average pain than males. Females further reported worst pain intensity (*U* (N_*Males*_ = 55, N_*Females*_ = 180) = 3652.5, *z* = −2.80, *p* < 0.01) and more interference of pain (*U* (N_*Males*_ = 54, N_*Females*_ = 180) = 3532.5, *z* = −3.12, *p* < 0.01), compared to males.

**TABLE 1:** Key demographic details and means (SD) for the DASS and recent pain experiences questionnaire.

DEMOGRAPHIC INFORMATION	
Sample Size Age	241 28.88 (10.83)
Gender	Males = 55 (23.23%) Females = 181 (74.85%) Prefer Not to Say = 5 (2.07%)
Nationality (Top 5)	British = 78 (32.37%) American = 54 (22.40%) Australian = 16 (6.64%) Canadian = 14 (5.81%) Other = 79 (32.78%)
History of Anxiety and/or Depression	Yes = 104 (43.2%) No = 127 (52.7%) Prefer Not to Say = 10 (4.1%)
First Language	English = 189 (78.4%) Other = 52 (21.6%)
QUESTIONNAIRE INDICES	MEAN (SD)
Depression (DASS-42)	15.8 (10.64)
Anxiety (DASS-42)	9.93 (9.0)
Stress (DASS-42)	14.23 (12.01)
Pain Frequency (last 3 months)	3.16 (2.92)
Pain Intensity (last 3 months)	2.53 (2.20)
Worst Pain Intensity (last 3 months)	4.58 (3.11)
Pain Interference (last 3 months)	2.48 (2.83)
Recent Pain Experiences (Composite)	12.68 (9.57)

*Theoretical ranges for the DASS-42/RPEQ sub-scales are outlined below: Depression (0-42), Anxiety (0-42), Stress (0-42), Pain Frequency (0-10), Pain Intensity (0-10), Worst Pain Intensity (0-10), Pain Interference (0-10).*

### Word Generation Task

In order to identify the most ambiguous scenarios, solutions provided by all participants were organized into three different categories; pain/pain-illness, non-pain/non-pain illness and difficult to define (DiD) (see Table 2 for category definitions). The percentage of solutions that fell into each category was then calculated. This provided insight as to those ambiguous scenarios that were open to multiple interpretations i.e., pain/pain-illness related and non-pain/non-pain illness related solutions. Scenarios whereby the proportion of solutions fell overwhelmingly (>75%) or underwhelmingly (<25%) into the pain/pain-illness related or non-pain/non-pain illness related categories (i.e., were not ambiguous as to being pain-related or otherwise) were removed. This ensured that only the most ambiguous scenarios were selected and resulted in the removal of 22 scenarios. One scenario, “Yesterday your bicycle was hit by a car. You will not be able to cycle for a while because the car broke your…” narrowly missed this criterion with 23.24% of the solutions falling into the pain category and 75.93% into the non-pain/illness category. That said, the responses reliably indicated one solution for the pain/illness category; that is, the word “Leg” accounted for the majority of the pain responses. Hence, the decision was taken to include this scenario, resulting in 20 scenarios being included in the final stimulus set for validation.

**TABLE 2:** Definitions used to categorize participant responses to the Word Completion Task.

Pain/Pain-Illness Definition	Non-Pain/Non-Pain-Illness Definition	DiD Definition
This category includes any word(s) or phrases that are indicative of immediate bodily harm (i.e., injury) or longer-term bodily harm (i.e., potential illness) to oneself or others, in the context of the ambiguous scenario. All professions associated with illness, disease and pain are included in this category (e.g., Dentist, Doctor, Optometrist etc.). Illnesses of an emotional and/or psychological nature (e.g., anxiety, depression) are not included in this category. Example: “You drop the kitchen knife onto the floor, it *stabs* your foot”	This category includes any word(s) or phrases that have no connection with immediate bodily harm (i.e., injury) or longer-term bodily harm (i.e., potential illness) to oneself or others. This category includes emotion-related words with positive/negative valence (e.g., Happy, Angry) and/or social-threat words (e.g., Embarrassed) in the context of the ambiguous scenario. Illnesses of an emotional and/or psychological nature (e.g., Anxiety/Depression) are included in this category). Example: “A bee lands on you and *touches* your hand”	This category includes any word(s) or phrases where: The word usage is unclear such that the word or phrase could be interpreted as fitting into more than one category The word(s)/phrases offered do not make sense in the context of the ambiguous scenario. Example: “You begin to breathe heavily. Your chest is quickly going up and down. You are *dead*”

### Word Generation Task: Inter-Rater Reliability

To ensure the main authors (DG) categorization of responses generated by the participants in the Word Generation Task was consistent with the definitions provided, two authors (FM/PS) categorized responses to a sub-set (20%) of the ambiguous scenarios. Initially, inter-rater agreement with DG ranged from 79.41% (FM) to 77.81% (PS). However, following meeting and refinement of the definitions (e.g., addition of professions text to the Pain/Pain-Illness definition), 100% agreement was observed across all three raters.

### Word Generation Task: Final Stimulus Set

A list of stimuli comprising the final scenario set for the Word Generation Task is presented in Table 3. In cases whereby the original pain/pain-illness and non-pain/non-pain illness solutions did not match the most popular answers generated by participants to these scenarios, the offered solutions for each scenario were changed to reflect this. Scenarios labeled **“OLD”** (*n* = 5) reflect those taken from previous research without changed solutions, **“OR”** (*n* = 1) reflects scenarios taken from previous research with changed solutions, **“N”** (*n* = 8) reflects new scenarios (i.e., those generated for purposes of the present study) without revision, and **“NR”** (*n* = 6) reflects new scenarios with revision. [For reference the most popular pain/pain-illness and non-pain/non-pain illness answers generated by participants for each scenario is also included].

**TABLE 3:** Word generation task: final stimulus set.

	Status	Most Popular Pain/Pain-illness solution	Most Popular Non-Pain/ Non-pain illness solution
A ball hits you in the face. You look in the mirror and see your face is covered in…	OLD	(Blood)	(Mud)
You teach your child how to cut mushrooms on a polystyrene plate. You become distracted and notice they have cut through their…	OLD	(Finger)	(Plate)
You use scissors to cut out a picture from a piece of paper. Suddenly, your hand slips and you cut into…	NR	(Your finger)	(The picture)
Your friend Jenny is brushing her hair; it is really messy. When it gets stuck in a tangle, she…	N	(Winces)	(Swears)
You sit down at a team meeting and accidentally trap your… under the chair.	NR	(Foot)	(Bag)
You are playing football and your friend tackles you. You feel…	N	(Hurt)	(Angry)
You notice a red stain on your shirt. You are worried the stain won’t come out because it is…	NR	(Blood)	(Wine)
You open a cupboard and a tin of baked beans falls out and hits…	NR	(Your foot)	(The floor)
You are playing sports with your brother; he runs into the house crying because you kicked the ball into…	NR	(His face)	(The neighbor’s garden)
You see your neighbor close her car door and grimace. This is because she shut her … in the door.	N	(Fingers)	(Dress)
Your mother receives the health-practitioner test results she has been waiting for. Your mother is crying because she has received…	N	(Bad news)	(Good news)
You drop the kitchen knife onto the floor, it… your foot.	N	(Hits)	(Misses)
The wind blows a tile from your roof. It hits your…	NR	(Head)	(Car)
A bee lands on you and… your hand	N	(Stings)	(Tickles)
You slip and fall on some ice when running to catch the bus, you feel…	N	(Hurt)	(Embarrassed)
You go indoors after sunbathing. Your skin feels…	N	(Burned)	(Hot)
Yesterday your bicycle was hit by a car. You will not be able to cycle for a while because the car broke your…	OLD	(Leg)	(Bike)
You make an appointment to see your doctor to discuss your test results. You think the results will show you are.	OLD	(Sick)	(Healthy)
It is 10am on a Monday and you are still in bed. You are at home because you have a…	OLD	(Cold)	(Day off)
When you wake up your eyes are swollen and it’s difficult to open them. This is due to.	OR	(Allergies)	(Crying)

*KEY: OLD = scenarios taken from previous research without revision; OR = scenarios taken from previous research with revision (changed solutions); N = new scenarios without revision; NR = new scenarios with revision (changed solutions).*

### Likelihood Rating Task

Data were transformed to calculate the total number of participants who rated the pain/pain-illness solution (or the non-pain/non-pain illness solution) as the most likely to complete each scenario. A score of “1” was assigned to the participant solution rated as most likely to complete the scenario and a score of “0” was assigned to the participant solution that was rated as least likely to complete the scenario; this enabled the identification of the stronger of the two endorsements. Below is an example of a participant’s response to a scenario:

“*A ball hits you in the face. You look in the mirror and see your face is covered in*…”

Pain/pain-illness solution: **Blood**
Participant Likelihood Percentage: **100%**
Non-pain/non-pain illness solution: **Mud**
Likelihood Percentage: **25%**

In this case, the pain/pain illness solution (i.e., Blood) is assigned a score of “1” and the non-pain/non-pain illness solution (i.e., Mud) is assigned a score of “0” because the participant has rated the pain/pain-illness solution as most likely to complete the scenario. Scores were then summed across all participants for each solution and converted into a percentage. In cases where participants rated the pain/pain-illness related and non-pain/non-pain illness related solutions as equally likely to end the sentence for the scenario (i.e., 50% and 50%, respectively), this data was removed and excluded from the final percentage calculation.

Scenarios were then selected based on two stages. First, scenarios whereby over 25% and under 75% of participants had chosen the non-pain/non-pain illness related solution to complete the scenario were selected for. This removed 14 scenarios for which participants were either very likely to choose the pain/pain-illness solution (i.e., <25% non-pain/non-pain illness choice) or very likely to choose the non-pain/non-pain illness solution (i.e., > 75% non-pain/non-pain illness choice); and so not ambiguous as to pain-related or otherwise. This left a sample of **28** scenarios for validation.

### Likelihood Ratings: Reliability Analyses

A series of analyses were performed on the likelihood ratings data for the remaining 28 ambiguous scenarios. Each analysis was conducted with the full set of 28 remaining scenarios to produce an optimal number of robust scenarios. No analyses were undertaken for the control scenarios.

### Likelihood Ratings: Forced-Choice Data Analysis

First, reliability analyses were carried out by using Cronbach’s Alpha on the Likelihood Ratings Data for the pain/pain-illness solutions for the 28 scenarios. These revealed the scenarios to have good internal consistency (α = 0.881). However, several scenarios had item-total correlations below optimal (*r* < 0.2). Sequential removal of four scenarios improved item-total correlations, with the remaining 24 scenarios correlating well with the total scale to an acceptable degree (lowest *r* = 0.33; α = 0.882).

The non-pain/non-pain illness solutions also possessed good internal consistency (α = 0.854). However, several scenarios had inter-item correlations below optimal (*r* < 0.2) suggesting they should be removed. Removal of one scenario improved the overall internal consistency (α = 0.856) of the scenarios. The removal of 3 further scenarios that had item-total correlations below *r* < 0.2 did not improve the item-total correlations or the alpha value returned. Consequently, these items were not removed.

Thus, the Likelihood reliability analyses indicated 23 ambiguous scenarios were fit for purpose.

### Likelihood Ratings: Forced-Choice Data

To obtain a measure of internal consistency for the pain/pain-illness solution data for the 28 scenarios, the Kuder-Richardson Formula 20 (KRF-20) was used, as data was dichotomous. Overall, the 28 scenarios had acceptable internal consistency (α = 0.65). However, multiple scenarios had item-total correlations below optimal (*r* < 0.2). Sequential removal of 9 scenarios improved overall internal consistency (α = 0.74). Deletion of further scenarios with correlations of *r* < 0.3 did not affect the alpha level returned, consequently these scenarios were not removed/.

The KRF-20 was also used to analyse the non-pain/non-pain illness solution data for the remaining 28 scenarios. Overall, the scenarios had acceptable internal consistency (α = 0.64). However, multiple scenarios had item-total correlations below optimal (*r* < 0.2). Sequential removal of 9 scenarios improved overall internal consistency (α = 0.72). Deletion of further scenarios with correlations of *r* < 0.3 did not affect the alpha level returned, consequently these scenarios were not removed.

Taken together, the reliability and KRF-20 analyses highlighted 9 scenarios as problematic. These included the 5 identified in the Cronbach’s Alpha analyses as problematic. Thus leaving 19 ambiguous scenarios. However, one further scenario was also removed due to the pain/pain illness solution of “fearful” being difficult to categorically define as pain-related (vs. anxiety related) according to our definitions. Therefore, this scenario was also removed resulting in the second stimulus set comprising 18 scenarios.

### Likelihood Ratings Task: Final Stimulus Set

A list of scenarios comprising the final set pertaining to the Likelihood Ratings Task is presented in Table 4 below. The researcher solutions for the pain/pain-illness and non-pain/non-pain illness categories are also provided. Scenarios labeled **“OLD”** (i.e., 8 out of 18) reflect those obtained from previous research, scenarios labeled **“N”** (i.e., 10 out of 18) reflect those developed for purposes of the present study.

**TABLE 4:** Likelihood ratings task: final stimulus set.

	Pain/Illness Solution	Non-Pain/ Illness Solution	Status
You wake up and notice how you feel today. You feel…	Sore	Refreshed	N
A ball hits you in the face. You look in the mirror and see your face is covered in…	Blood	Mud	OLD
Your cousin visits the doctor to get his test results back. His growth is…	Cancerous	Benign	OLD
You teach your child how to cut mushrooms on a polystyrene plate. You become distracted and notice they have cut through their…	Hand	Plate	OLD
You use scissors to cut out a picture from a piece of paper. Suddenly, your hand slips and you cut into…	Your Finger	The picture	N
You notice a red stain on your shirt. You are worried the stain won’t come out because it is…	Blood	Pen	N
You open a cupboard and a tin of baked beans falls out and hits…	Your head	The floor	N
You are playing sports with your brother; he runs into the house crying because you kicked the ball into …	His face	The neighbor’s garden	N
You see your neighbor close her car door and grimace. This is because she shut her… in the door.	Fingers	Coat	N
Your mother receives the health-practitioner test results she has been waiting for. Your mother is crying because she has received…	Bad News	Good news	N
You drop the kitchen knife onto the floor, it … your foot.	Cuts	Misses	N
The wind blows a tile from your roof. It hits your…	Head	Car	N
A bee lands on you and … your hand.	Stings	Tickles	N
Yesterday your bicycle was hit by a car. You will not be able to cycle for a while because the car broke your…	Leg	Bike	OLD
Your Dad is driving you in the car. Suddenly your car hits the car in front of you. You are…	Hurt	Scared	OLD
You make an appointment to see your doctor to discuss your test results. You think the rest results will show you are …	Ill	Fine	OLD
It is 10am on a Monday and you are still in bed. You are at home because you have a …	Cold	Holiday	OLD
You begin to breathe heavily. Your chest is quickly going up and down. You are…	Asthmatic	Exercising	OLD

*Key: OLD = scenarios taken from previous research; N = new scenarios.*

### Control Scenarios: Data Analysis

The filler stimulus set comprising 20 scenarios were tested for ambiguity. Given the nature of these scenarios, the criteria applied to the ambiguous scenarios set for the Word Completion Task and Likelihood Ratings Task was deemed unsuitable. Instead, scenarios were removed based upon several criteria. This included: **(i)** if either of the two most popular solutions had positive or negative connotations; **(ii)** the scenario was not ambiguous (i.e., the second most popular answer was disproportionately selected, in that less than 10% of the sample generated this response); and **(iii)** the two most popular answers for one scenario were identical to a different scenario. After applying these criteria, 12 filler scenarios remained. Next, in cases where the two most popular solution(s) for the filler scenarios did not match the solutions initially generated by the researcher, the most popular solutions provided by participants were used in replacement. Of the 12 remaining scenarios, this led to 6 scenario solution changes.

### Control Scenarios: Final Stimulus Set

A list of the full filler scenarios is presented in Table 5. To enable use in likelihood rating style tasks (as well as word generation tasks), the top two solutions for each scenario are provided. All 12 filler scenarios were generated for purposes of the present study (i.e., none were obtained from previous research).

**TABLE 5:** Filler scenarios: final stimulus set.

	Top Filler Solution 1	Top Filler Solution 2
You watch the weather forecast on the TV. Tomorrow it is forecast to be a … day.	(Sunny)	(Rainy)
You receive a letter from your child’s head teacher. This was written using a …	(Pen)	(Computer)
You see some fish swimming in the water. They are swimming in a …	(Pond)	(Circle)
Your partner is late to an important meeting. This is because they forgot their …	(Phone)	(Keys)
You get home from work and realize you left the … on.	(Light)	(Oven)
After a long day, your grandmother likes to have a drink of …	(Tea)	(Wine)
You get distracted and when you return you realise you forgot to boil the…	(Water)	(Kettle)
During a chat, your younger sister tells you she wants to learn how to ride a …	(Bike)	(Horse)
You let your dog off the lead at the local park. Immediately your dog sprints to fetch a …	(Stick)	(Ball)
The postman brings you a delivery you had been expecting. You open the…	(Package)	(Box)
You look across the room and see your cat. He is sat on the…	(Sofa)	(Mat)
You arrive at the office to start the working day. You turn on the.	(Computer)	(Light)

### Recent Pain Experiences and Likelihood Ratings Task

Forty-three participants reported experiencing no pain in the preceding 3 months and therefore their scores on the RPEQ were transformed. That is, these participants were assigned a score of “0” for pain intensity and a score of “0” for interference and a score of “1” for frequency, in accordance with previous research (Heathcote et al., 2016).

Next, the relationship between recent pain experiences and likelihood ratings for pain/pain-illness and non-pain/non-pain illness solutions for all scenarios on the Likelihood Ratings task were assessed. There was a weak, significant, positive correlation between recent pain experiences and likelihood ratings for pain/pain-illness solutions (*r* = 0.164, *n* = 241, *p* = 0.005, *one-tailed*). There was also a weak, negative, non-significant correlation between recent pain experiences and likelihood ratings for non-pain/non-pain illness solutions (*r* = −0.086, *n* = 241, *p* = 0.09, *one-tailed)*.

Simple linear regression with a composite recent pain experiences score as the predictor variable and the likelihood ratings for the pain/illness solutions as the outcome variable revealed that participant’s recent pain experiences predicted likelihood ratings for the pain/illness related solutions (*F*(1, 240) = 6.61, *p* = 0.01) with an *R*^2^ of.027. So, recent pain experiences explained 27% of the variance in the likelihood ratings assigned to the pain/illness solutions. However, when the likelihood ratings for the non-pain/non-pain illness solutions were included as the outcome variable, no significant regression equation was found [(*F*(1, 240) = 1.78, *p* = 0.18) with an *R*^2^ of < 0.01]. In other words, recent pain experiences did not explain any variance in the likelihood ratings assigned to the non-pain/non-pain illness solutions.

## Discussion

The purpose of the present study was to validate a stimulus set of ambiguous scenarios that can be used to measure IBs in adults. However, two ambiguous stimulus sets, that have good internal consistency, were actually developed: a Word Completion Set and a Likelihood Ratings Set. Additionally, we also developed a set of 12 control scenarios that can be used with either task to avoid priming. Therefore, the two sets comprise 32 and 30 stimuli, respectively. Analyses revealed that participants who reported more recent pain experiences in the past 3 months were more likely to endorse the pain/pain-illness solutions for the scenarios presented in the Likelihood Ratings Task, providing evidence of a negative endorsement bias. The utility of these stimulus sets for pain-related research as well as treatment program efficacy evaluation will be discussed.

Previous research investigating IB has relied upon paradigms such as the Homophonic/Homographic response task (McKellar et al., 2003). These tasks include a small number of appropriate stimuli and can be influenced by stimulus word frequency rather than pain biases (Schoth and Liossi, 2017). The AIBT (Heathcote et al., 2016) was designed to address these limitations. However, it is constrained by a forced-choice response format and lack of validation in adult samples. Forced-choice response formats may not necessarily reflect the interpretations of each participant, questioning whether they are a suitable measure of all pain/pain-illness related IBs. In contrast, our stimulus sets support two response formats; forced-choice and free response and, additionally, are appropriate for adult populations. Moreover, as awareness of potentially threatening information can influence cognitive decision-making, such as whether individual’s attend to or avoid such information (Lapate et al., 2014; Hedger et al., 2015), the sole presentation of ambiguous pain-illness/non-pain illness scenarios may inadvertently influence participant responding (e.g., pain related responding may prime further pain related responding). Our additional integration of control scenarios helps to circumvent this priming, as well as confounds of order effects and demand characteristics.

Considering the forced-choice ambiguous scenario set, we have produced a scenario set containing 30 scenarios (18 ambiguous; 12 control) that can be used with adult populations in IB related research/treatment efficacy evaluation. In addition, we have further produced a “word generation” scenario set containing 32 scenarios (20 ambiguous; 12 control). This stimulus set arguably possesses greater utility in measuring pain related IBs, compared to forced-choice paradigms utilized previously, given the scenarios are open-ended, avoiding limitations/constraints associated with forced-choice paradigms (Schoth and Liossi, 2017).

Supplementary analyses of participants self-reported recent pain experiences and likelihood task solution ratings further revealed that participants’ who reported more recent pain experiences assigned a significantly higher likelihood rating to pain/illness related solutions compared to non-pain/non-illness solutions. These findings are in accord with previous IB research and theoretical models of pain (Van Ryckeghem et al., 2019). Heathcote et al. (2016) found that adolescents who catastrophized about pain and reported more recent pain experiences showed a tendency to endorse pain/illness related interpretations, rather than benign interpretations, of ambiguous situations. A finding that was later replicated with adolescent chronic pain sufferers (Heathcote et al., 2017). Lau et al. (2019) observed similar findings, with adolescents who reported moderate-to-high pain interference being more likely to endorse pain/illness interpretations across all interpretations compared with their non-interfering pain counterparts. Moreover, Chan et al. (2020) found adults with chronic pain displayed a negative endorsement bias for ambiguous scenarios pertaining to immediate bodily injury/long-term illness. Our findings are consistent with such previous research, demonstrating that adults with acute and/or chronic pain favor pain/pain-illness related interpretations of ambiguous information (Schoth and Liossi, 2016). Taken together, these findings provide validation of the stimulus sets (obtained from the Likelihood Ratings Data), and demonstrates they are fit for purpose to measure IBs in Adults.

Ambiguous scenarios are of critical importance to investigate pain related IBs more rigorously, and treatment program efficacy (An et al., 2020; Chan et al., 2020), as it remains unclear whether interventions that aim to re-train negative IBs in pain patients are effective. Cognitive Bias Modification for Interpretation (CBM-I) involves training pain patients away from a negative interpretation style that favors pain/illness interpretations, to a more adaptive interpretation style that favors neutral or even positive interpretations. An et al. (2020) recently developed an Interpretation Bias Modification Task for Pain (IMB-P), which presented ambiguous pictures that could be interpreted in an objective or pain-related manner. Participants were presented with two sentences, reflecting an objective (e.g., a person lays hands on their knee) or pain-related (e.g., a person lays hands on a sore knee) interpretation, and were asked to select the sentence that best described the picture. Participants allocated to the training group received positive feedback whenever they selected the non-pain (i.e., objective) sentence. Findings showed that chronic pain patients allocated to the training group showed less IB and negative emotions compared to the control group after a single session of training. This training also impacted attentional biases, with the training group gazing longer at neutral words compared to new affective words post-training, then they did prior to the intervention. Importantly, this study provides preliminary evidence to suggest that CBM-I or IBM-P paradigms that use ambiguous scenarios can modify cognitive biases and thus may possess clinical utility in pain management. However, it is important to note that the findings of An et al., may be limited given that IBs were measured via the Homographic Response Task and, as previously suggested, participant responding on such tasks can be influenced by stimulus word frequency rather than pain biases. Hence, as IBs are now the focus of some pain intervention treatments, it is critical that researchers have a variety of tools necessary to ensure accurate measurement of such biases; this would include utilizing the Ambiguous Scenario stimulus sets we have produced in their repertoire. For example, alongside paradigms including Ambiguous Words (e.g., Sentence Generation Task, Word Stem Completion Task) and Ambiguous Images (e.g., Incidental Learning Task).

### Methodological Considerations

A limitation of the present study was the inability to perform reliability for the scenarios generated via the Word Generation Task, as participants were not constrained to a pre-determined list of interpretations. However, this is also a strength of the present study; that is, it enabled participants to generate novel solutions. Inter-rater reliability was not considered problematic as the agreement pertaining to the categorization of participants responses was high, averaging 79.41% (FM) to 77.81% (PS) before discussion, and 100% after. This not only shows good validity of our categorizations; but also provides evidence of criterion validity for the Word Generation Task. A second limitation that could be levied against the present research concerns the use of word stimuli. Word stimuli are argued to possess lower ecological validity than pictorial stimuli (e.g., pain-related facial expressions, Schoth and Liossi, 2017), given they require cognitive processing. This is of particular importance when measuring attentional biases given that attentional biases are debatably pre-cognitive biases (see Gaffiero et al., 2019; Maratos and Pessoa, 2019). Arguably interpretation biases are cognitive processes and therefore word stimuli in these cases are less problematic. Nevertheless, ecological validity of stimuli is always an issue a researcher should consider. Indeed, while IB can be quantified via valence orientation (e.g., positive vs negative), other factors also important to consider include rigidity and adaptiveness in relation to context (Mehu and Scherer, 2015; Van Ryckeghem et al., 2019). For example, interpretation patterns related to acute versus chronic pain may differ in their adaptiveness and thus be viewed differentially. Relatedly, distinguishing between anticipation and attribution (i.e., preventing immediate harm vs. appraising implications of chronic pain) could have important implications for the ecological validity of stimulus sets and the future development of pain-related stimulus sets. To expand, scenarios based on immediate pain anticipation (e.g., a knife hitting or otherwise your foot) may be quite different to those associated with pain attribution (e.g., test results from your doctor). For example, it is considered adaptive to avoid events with the potential to cause immediate bodily injury. In comparison, appraising a medically explained, persistent pain as immediately threatening, subsequently fueling fear-avoidance behaviors, would be relatively less adaptive. Hence, developing paradigms/stimuli that can distinguish between scenarios based on anticipation and attribution would enable us to examine how these further factors influence interpretation biases in acute and/or chronic pain populations. This serves not only as a limitation of the current study and the stimulus sets produced, but also that of *all* previous pain related IB research, and thus should be a consideration of future studies in this field.

### Future Research

In addition to the further exploration of the differential effects (or otherwise) of anticipation and attribution within stimulus sets, cognitive-affective models of pain suggest that negative (pain or illness) interpretations may also influence biased attending to pain (Van Ryckeghem et al., 2019). To test these theoretical assumptions, research is needed to investigate the relationship between interpretation and attentional biases. Previous research has typically investigated these biases in isolation. However, to further knowledge, understanding and theory in the field of pain, multiple biases should be measured within the context of a single study. For example, the scenarios developed as part of the present study could also be incorporated with other paradigms to examine the role recall biases play in acute/chronic pain. Here, for example, participants could be presented with both pain/pain-illness and non-pain/non-pain illness solutions for the Likelihood Ratings Task and investigation of which they recall at a later date probed. Recalling more solutions that reflect a pain/pain-illness interpretation, as opposed to a non-pain/non-pain illness interpretation, would provide evidence to suggest pain memory biases. Until now, this has not been possible due to the lack of validation of ambiguous scenarios in adult samples. Importantly, this brief future research example highlights the adaptability and potential utility of the ambiguous scenarios and sets developed in the present study to advance knowledge regarding combined cognitive biases in pain.

Secondly, pain catastrophizing appears to play a central role in the development and maintenance of chronic pain related disability (Eccleston and Crombez, 1999; Vlaeyen and Linton, 2000; Giusti et al., 2020; Varallo et al., 2021) and appears to be associated with IB in both clinical and control samples (Vancleef et al., 2009; Khatibi et al., 2014, 2015). Considering this, future studies could test this association and evaluate whether interventions aimed at reducing pain catastrophizing (e.g., Cognitive Behavioral Therapy) influence IBs.

## Conclusion

The aim of the present study was to validate a series of ambiguous scenarios that can be interpreted in a pain/pain-illness and/or non-pain/non-pain illness related fashion. Importantly, two separate stimulus sets that allow for two response formats, forced-choice and free response, were developed for use in pain/pain-illness IB research to address limitations of previous research. For the forced-choice likelihood scenario task, supplementary analyses revealed that adults who reported more recent pain experiences over the past 3 months were more likely to assign a higher likelihood rating to the pain/pain-illness solutions, lending support to previous pain-related-bias research in this area (Heathcote et al., 2016, 2017; Lau et al., 2019). As such, the current study provides two new stimulus sets that can be utilized to measure pain/illness related IBs in adults. Future research should examine the utility of ambiguous scenario tasks within the context of interventions, such as CBM-I, which may prove effective in training adults with a pain/illness interpretation style to adopt a more adaptive interpretation style; which may ultimately help with pain coping. This is especially important given the direct effects of COVID-19 on people with Chronic Pain, including increased pain due to physical inactivity, delays to and/or the stopping of treatment and opioid-overuse (El-Tallawy et al., 2020; Javed et al., 2020). Hence, there are ever increasing calls for new and effective ways of pain management (Karos et al., 2020).

## Data Availability Statement

The raw data supporting the conclusions of this article will be made available by the authors, without undue reservation.

## Ethics Statement

The studies involving human participants were reviewed and approved by Human Sciences Research Ethics Committee (HSREC) - University of Derby. The patients/participants provided their written informed consent to participate in this study.

## Author Contributions

DG, FM, and PS incepted the study. DG, FM, and PS designed the study stimuli. DG conducted the study. DG with support from PS, VS, and FM analyzed all study data. DG with support from FM produced the first draft of the manuscript, which VS and then PS provided feedback on. All authors discussed the results and commented on the manuscript.

## Conflict of Interest

The authors declare that the research was conducted in the absence of any commercial or financial relationships that could be construed as a potential conflict of interest.

## Publisher’s Note

All claims expressed in this article are solely those of the authors and do not necessarily represent those of their affiliated organizations, or those of the publisher, the editors and the reviewers. Any product that may be evaluated in this article, or claim that may be made by its manufacturer, is not guaranteed or endorsed by the publisher.
